# Cerebellar BOLD signal during the acquisition of a new lexicon predicts its early consolidation

**DOI:** 10.1016/j.bandl.2015.07.005

**Published:** 2016-10

**Authors:** Elise Lesage, Emma L. Nailer, R. Chris Miall

**Affiliations:** a School of Psychology, University of Birmingham, UK; b Neuroimaging Research Branch, National Institute on Drug Abuse, National Institutes of Health, Baltimore, MD, USA; c School of Education, University of Birmingham, UK

**Keywords:** Cerebellum, Language, Vocabulary, Learning, Lexicon, fMRI, Word association, Non-motor

## Abstract

Cerebellar contributions to language are presently poorly understood, but it has been argued that the cerebellar role in motor learning can be extended to learning in cognitive and linguistic domains. Here, we used fMRI to investigate whether the cerebellum is recruited in mapping novel words onto existing semantic concepts. On separate days, participants performed a Basque vocabulary learning task and a control English synonym task in the MRI scanner. Learning-related BOLD activity was found in left inferior frontal gyrus, bilateral insula, pre-SMA, left superior parietal cortex, right caudate, the right cerebellar vermis and right cerebellar Crus II. The extent to which the cerebellar regions, but not the cerebral areas, were recruited during learning correlated positively with participants’ off-line improvement in performance after the learning task. These data provide evidence for a cerebellar role in lexical learning, and suggest that the right cerebellum may contribute toward consolidation of lexico-semantic associations in the language network.

## Highlights

• We measured learning of Basque words in the MRI scanner.
• A control task involved identification of English synonyms.
• The right cerebellum was recruited during the learning of new words.
• Cerebellar BOLD response predicted individual improvement after the task.
• Our results suggest that the right cerebellum contributes to lexical learning.

## 1 Introduction

In recent decades it has become increasingly clear that the cerebellum plays a role in linguistic, cognitive and emotional processes as well as motor processes (Schmahmann, 2010, Stoodley et al., 2012, Strick et al., 2009). Cerebellar regions that contribute to these non-motor processes are distinct from those that are engaged in motor control, and they project to higher-order areas of the neocortex such as prefrontal and posterior parietal regions (Buckner et al., 2011, Kelly and Strick, 2003, Middleton and Strick, 2001, O’Reilly et al., 2010). Patients with right cerebellar lesions can present with problems with verbal fluency, lexical access, and grammar (De Smet et al., 2013, Fiez et al., 1992, Gebhart et al., 2002, Scott et al., 2001), though left and midline damage has also been associated with language problems (Cook et al., 2004, Fabbro et al., 2000, Murdoch and Whelan, 2007, Riva and Giorgi, 2000). Neuroimaging studies in healthy subjects consistently find right posterolateral cerebellar activation (specifically right Crus I and Crus II) in language contrasts (E et al., 2012, Stoodley and Schmahmann, 2009a). Thus, clinical and neuroimaging evidence implicate predominantly the right posterolateral cerebellum, which is connected to left-lateralized higher-order cerebral language regions, in language processing (Mariën, Engelborghs, Fabbro, & De Deyn, 2001).

The cerebellum is a highly plastic structure, and its role in acquisition and adaptation of motor skills is well-established (Jenkins et al., 1994, Raymond et al., 1996). However, the precise function of cerebellar non-motor regions remains unclear. It has been repeatedly argued that cerebellar areas that are active during cognitive and linguistic tasks likely perform similar functional operations (i.e. skill acquisition and adaptation) as cerebellar regions involved in movement (Bloedel, 1992, Ito, 2008, Miall, 2003, Ramnani, 2006). If all regions of the cerebellar cortex do indeed perform common operations, then non-motor posterolateral regions of the cerebellum should be recruited in the acquisition of abstract linguistic and cognitive rules or associations. In support of this notion, neocerebellar structures are activated when participants acquire abstract, rule-based associations (Balsters and Ramnani, 2011, Balsters et al., 2013). To date, no study has investigated whether the right posterolateral cerebellum is also recruited during the acquisition of semantic associations, such as the mapping of a novel word onto an existing semantic concept. However, BOLD activation of the right lateral cerebellum has been reported in studies of word learning. These studies report right cerebellar activity in implicit learning tasks (Breitenstein et al., 2005), in comparing the familiarity of novel versus previously learned words (Davis, Maria, Betta, Macdonald, & Gaskell, 2009), or in relation to vocabulary training over several weeks (Raboyeau et al., 2004).

The present paper investigates a possible cerebellar role in the acquisition of a new lexicon. Learning a new lexicon is a necessary first step in learning a new language. Near-instantaneous word learning has predominantly been studied in young children (Dollaghan, 1985), where the EEG response to novel words can match that of familiar words after as little as 14 min of training (Shtyrov, Nikulin, & Pulvermüller, 2010), but rapid vocabulary learning over several hours is also documented in adults (McLaughlin et al., 2004, Osterhout et al., 2008). The acquisition of a novel lexicon depends on the hippocampus (Gabrieli et al., 1988, Gooding et al., 2000), and it is thought that after an initial hippocampus-dependent phase, representations are transferred to various cortical regions which then store the semantic long-term memory (Battaglia, Benchenane, Sirota, Pennartz, & Wiener, 2011). The latter process, consolidation, can refer to two distinct processes that occur over different timescales (Dudai, 2004). The first, synaptic consolidation, is thought to take place in the medial temporal lobe over the minutes to hours after the training task. The second is system-level consolidation, which is thought to involve the transfer of hippocampal memories to the neocortex, be sleep-dependent, and to occur over days to even months (Dudai, 2004). However, in recent years it has become clear that system-level consolidation can occur over much shorter intervals than previously thought (Tse et al., 2007). Moreover, neuroimaging studies of memory tasks and incidental language learning tasks show the early recruitment (i.e. before sleep) of neocortical areas during encoding of verbal materials and during incidental vocabulary learning. These extra-hippocampal areas consistently involve the left inferior frontal gyrus (Buckner et al., 1999, Kapur et al., 1994, Kapur et al., 1996), but also include the left dorsolateral prefrontal cortex, left temporal cortex, left angular gyrus, and the right cerebellum (Breitenstein et al., 2005, Davis et al., 2009, Dolan and Fletcher, 1997). Thus, consolidation at timescales consistent with synaptic consolidation can involve the neural structures associated with system-level consolidation. We do not address this distinction, but behaviorally define offline performance improvement occurring over the first 20 min after the learning task as early consolidation. We refer to early consolidation rather than synaptic or systems-level consolidation, because we make no assumptions about the underlying mechanism, or the recruitment of non-hippocampal structures. Thus far, very few neuroimaging studies have investigated the neural correlates of early stages of explicit vocabulary learning and consolidation (Shtyrov, 2012). In this study, we therefore use fMRI to look at haemodynamic activity related to a vocabulary learning task inducing rapid acquisition of a new lexicon.

Participants learned 25 Basque translations for English words in a vocabulary learning task (second language task) in a block-design fMRI task. Activations during this lexical learning task were compared with activations during a closely matched control task (first language task) performed on a different day. In light of the evidence that the right posterolateral cerebellum may be part of a “common semantic network” (Vandenberghe, Price, Wise, Josephs, & Frackowiak, 1996), and in light of the role of the cerebellum in higher-order rule learning (Balsters et al., 2013), we hypothesize that the right lateral cerebellum will be amongst the regions that are involved in the acquisition of a new lexicon. In addition, we test the hypothesis that individual differences in the BOLD activation in the cerebellum will predict performance gains during the task and performance gains after the task, as a marker of early consolidation processes (Albert, Robertson, & Miall, 2009).

## 2 Methods

### 2.1 Participants

Participants were 15 right-handed native English speakers (mean age 24 years, 5 male). None of them spoke the Basque language or Spanish, or had spent time in the Basque country prior to the experiment. Participants were screened with a standard MRI screening questionnaire (http://prism.bham.ac.uk/downloads/MRI_screening_form.pdf). This study was approved by the Birmingham University Imaging Centre (BUIC) Ethics Programme. Written informed consent was obtained from each participant prior to the experiment and participants were compensated financially for their time.

### 2.2 Design and procedure

**Fig. 1:**
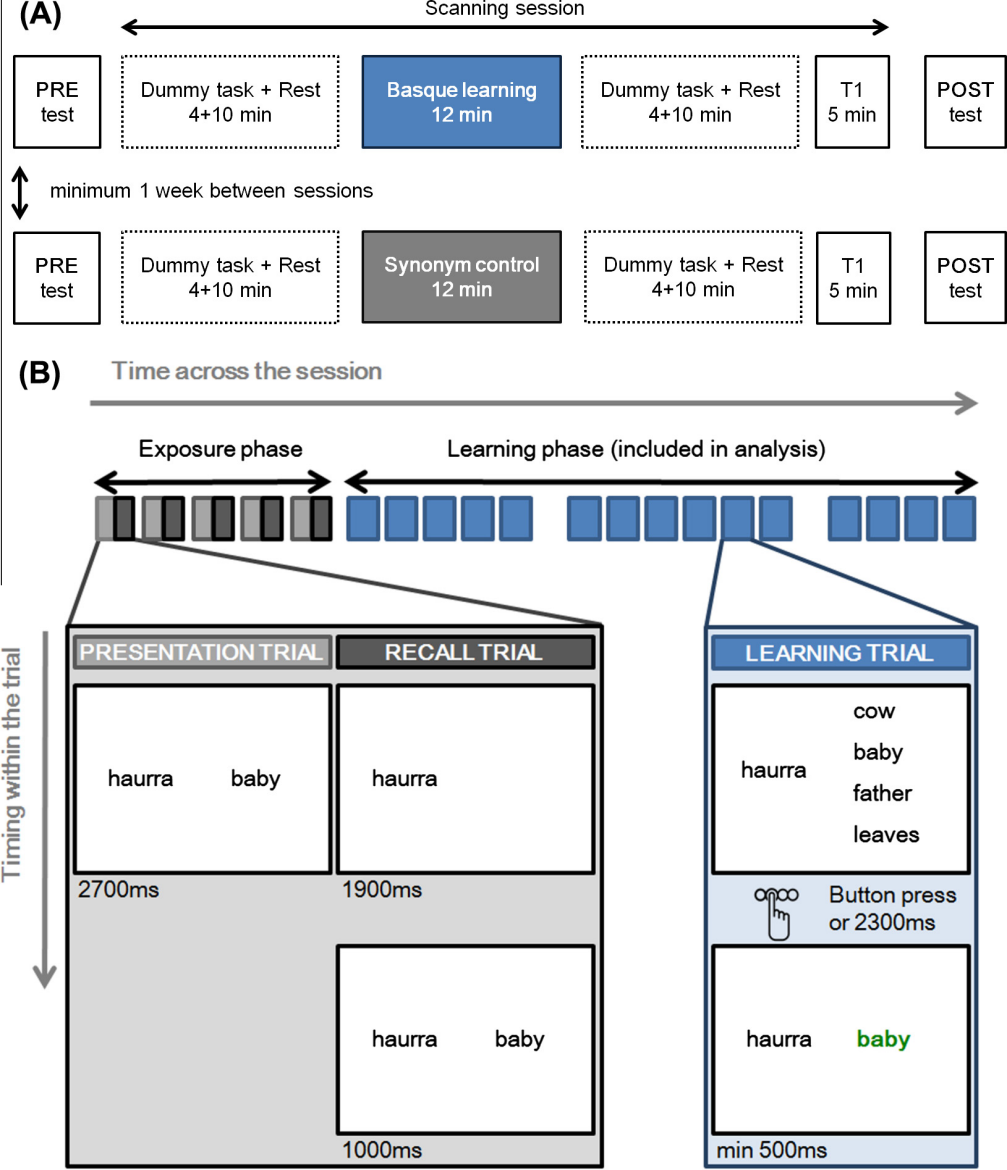
(A) Time line of the scanning sessions. Each participant participated in both sessions, with a minimum of 1 week between sessions. The order of the sessions was counterbalanced between participants. (B) Learning task: stimulus display, block structure, trial types and their timing. The bar at the top of the figure illustrates the occurrence of the various block types throughout the experiment. Gaps between the blocks indicate null blocks. The exposure phase consisted of 5 blocks (32 s) each of which consisted of 5 presentation trials (i.e. within each light gray box) followed by 5 recall trials (dark gray boxes). The bottom panels show the on-screen display (white boxes) and their time-course (with time within the trial represented vertically). Each learning block (blue, 18.2 s) consists of 5 learning trials.

Each participant was scanned during two sessions; one session where a Basque vocabulary (second language) learning task was performed, the other where a control English synonym (first language) task was performed (see Fig. 1A). We chose Basque as the second language because it uses the Latin alphabet like English, but is not an indo-European language. Basque is unrelated to the languages our population would most likely have been exposed to, such as English, French or German. We thus minimized the chance that participants would have prior knowledge of the items. The order of the sessions was counterbalanced between participants, with 7 participants performing the control session prior to the Basque session. Sessions were at least one week apart. During each session 25 Basque words or English synonyms were repeatedly presented (see Section 2.3).

Before scanning, a multiple choice questionnaire (‘pre-test’) assessed prior knowledge of the words. The scanning session lasted about 50 min and included a 10 min resting state scan before and after the learning task, which lasted 12 min (see Fig. 1A). Each of the resting state scans was preceded by a dummy task, where participants observed dynamic point light displays of human biological motion or scrambled version of these stimuli (see also Albert et al., 2009). No images were acquired during the dummy tasks. The resting state data is not considered in the present paper. Following the second resting state block, a T1 structural scan was acquired. After scanning (about 20 min after the learning task), subjects filled in a second multiple choice questionnaire (‘post-test’). Participants who did the control session first did not perform the post-test immediately after the control scanning session and instead these 8 participants filled in both post-tests at the end of the second (Basque) session. This was done to ensure that participants were unaware that there would be a post-test after the Basque learning task, and to thereby avoid that they would be ‘studying’ during the remainder of the scanning session. Therefore, none of the participants had reason to expect a post-test after the MRI experiment in the Basque session. In this study, the 12-min learning task is an explicit learning task, as participants were made aware of the to-be-learned associations, received feedback on their responses, and (presumably) were making a conscious effort to learn these associations. By contrast, any performance improvement after the task is very likely the result of implicit processing.

### 2.3 Task

#### 2.3.1 Basque learning task

The Basque learning task consisted of 5 exposure blocks, followed by 15 learning blocks. Each of the 5 exposure blocks introduced 5 novel Basque words, so 25 Basque words were learnt in total (see supplementary materials for the items used). An exposure block lasted 32 s and consisted of two parts: 5 presentation trials followed by 5 recall trials. In each presentation trial, a Basque word was presented alongside its English translation for 2700 ms. During a recall trial, a Basque word was presented for 1900 ms, after which the translation appeared alongside the Basque word for 1000 ms. This phase resembled a ‘flashcard’ revision procedure. For the different trial types, see Fig. 1B.

The exposure phase was followed by 15 learning blocks that each lasted 18 s. Each learning block consisted of 5 trials and was set up as a multiple choice test. During a learning trial, a Basque word was presented on the left, along with 4 English words on the right, one of which was the correct translation. Participants had 2300 ms to decide and to press one of 4 buttons on an MR-compatible response box. As soon as participants had pressed a button, or after 2300 ms if they had not responded, the correct translation was presented for the remainder of the trial (see Fig. 1B). The correct translation was presented in green for a minimum of 500 ms, regardless of the accuracy of the response, and was meant to provide a further learning opportunity rather than merely give feedback on the performance. Each word was repeated 3 times over the course of the 15 learning blocks. The responses to these multiple choice questions were used for behavioral analysis. The order of the words was pseudorandom, with the requirement that the same word was never repeated in the same block. The pseudorandom order of the items served to prevent participants from learning the order of the items. The multiple choice alternatives were different at each presentation of a given word. The correct translations were presented at a different place amongst the four alternatives (corresponding to a different button) at each repetition and were also used as distracters in trials with a different Basque word. All block onsets were temporally jittered with regards to the onset of the TRs (with an added delay from a uniform distribution ranging from 0 ms to 3000 ms). There was always at least 9 s (3TRs) between blocks. Two null blocks were also included to further improve statistical efficiency by increasing the number of volumes available to estimate the implicit baseline (see Fig. 1A; Friston, Zarahn, Josephs, Henson, & Dale, 1999). Null blocks lasted 18 s and entailed the presentation of a blank screen. As such, they were indistinguishable from the rest periods between the blocks and appeared as longer gap between two learning blocks. Over the course of the learning task, 22.2% of time was spent on exposure blocks, 37.9% on learning blocks, and 39.9% on rest intervals between blocks. These rest intervals between blocks was used to estimate the implicit baseline in the first level analysis.

#### 2.3.2 Control task

The control task was identical to the Basque task, except that instead of Basque–English word pairs, 25 English synonym pairs were used. Pilot tests in a different group of subjects indicated that the synonyms were likely to be known to all participants. In the present participant sample, the 100% correct performance on the pre-test confirmed that the items were indeed known. The structure of the control task was the same as that of the Basque task. First, synonym pairs were presented during 5 exposure blocks, that each consisted of 5 presentation trials and 5 recall trials. Then, 15 learning blocks were presented, each consisting of 5 trials where participants made multiple-choice type responses. Block timing, including the two null blocks, was identical between the Basque and the Control session. Thus, task procedure, timing, and sensory and motor demands of the two task sessions were identical. The only difference between the tasks was that the semantic mapping of the control pairs was known before the task, whereas that of the Basque pairs was not known before the task.

#### 2.3.3 Practice task

Prior to the study, participants performed a laptop-based training version of the different blocks, to familiarize themselves with the task they would be performing inside the scanner. These training blocks used different word stimuli from the ones in the main task.

### 2.4 MRI acquisition

Images were acquired on a 3T Philips Achieva scanner with an 8-channel head coil at the Imaging Centre at the University of Birmingham (https://www.buic.bham.ac.uk/). Functional images were obtained with an ascending EPI sequence (TR = 3 s, TE = 32 ms, 52 axial slices (no gap), voxel size 3 mm^3^ isotropic FOV 240 × 240, flip angle = 85°). A high-resolution T1-weighted structural scan was acquired at the end of each session (3D TFE, TR = 8.4 ms, TE = 3.8 ms, 175 sagittal slices, voxel size 1 mm isotropic FOV 288 × 234 × 175, flip angle 8°). Pulse oximetry and breathing traces were recorded using Philips-integrated systems for physiological monitoring. Recording these traces allowed us to regress out cardiac and respiratory signals that might confound the BOLD signal, particularly around the cerebellum and brainstem (Schlerf, Ivry, & Diedrichsen, 2012).

## 3 Analysis

### 3.1 Behavioral analysis

Task performance was analyzed to ensure that participants’ performance increased during the Basque session, and that performance was at a very high level and did not increase further in the control session. Performance was assessed outside of the scanner during the pre-test and the post-test, as well as during the learning phase of the scanner task. The learning phase consisted of 75 multiple-choice questions over 15 blocks, and the response to these questions served as the performance measure. Each of the 25 items was presented three times over the course of the scanning session. It should be noted that these three testing points were not evenly spaced over the course of the task, as the item order was pseudorandom to avoid participants learning the order of the items (see Section 2.3). Thus, performance was assessed at 5 time points: during the pre-test and the post-test (outside the scanner), and 3 times during the learning phase of the fMRI task. Due to a technical problem, the behavioral data of one subject during the scanning session were lost, and therefore are not included in the behavioral analysis. Because the behavioral data violate the assumptions of homogeneity of variances and of normality, parametric tests were inappropriate. To test for a time-by-condition interaction, participants’ scores for the Basque words and the synonyms were subtracted, and a Friedman’s ANOVA was conducted on these difference scores to assess change over time. This analysis was followed by post-hoc Wilcoxon rank tests to test for differences between each pair of time points. Analyses were carried out using SPSS.

### 3.2 FMRI analysis: task blocks

Preprocessing. Imaging analyses were carried out in SPM8 (http://www.fil.ion.ucl.ac.uk/spm/). Raw images for each session were motion-corrected, slice-time corrected, and co-registered to the mean image before first level analysis. First level analysis was performed on images in subject-specific space. Further processing was performed on the contrast images created during the first level analysis. The analysis pipeline was segregated for the cerebellum and the rest of the brain. Standard normalization as implemented in SPM8 is suboptimal for subcortical regions such as the cerebellum (Klein et al., 2009). To overcome this, the cerebellum was analyzed separately using the SUIT toolbox in SPM8 (Diedrichsen, Balsters, Flavell, Cussans, & Ramnani, 2009). First, participants’ cerebella were isolated from the T1 images and normalized to the SUIT template (Diedrichsen, 2006). Contrast images from the individual’s analyses were then normalized to the cerebellar template, as well as to the SPM8 EPI template for whole-brain analysis. Finally, images were smoothed with an 8 mm full width at half maximum (FWHM) Gaussian smoothing kernel before entering second level analysis. BOLD signals around the brainstem and cerebellum can be vulnerable to confounding physiological signals, but these can be controlled for by regressing out heart rate and breathing traces in the GLM model (Schlerf et al., 2012). The PhLEM toolbox in SPM (Verstynen & Deshpande, 2011) was used to convert heart rate and breathing traces into SPM regressors with a CETROICOR method (Glover, Li, & Ress, 2000).

First level analysis. The first level general linear model of each session included two regressors of interest: one that modeled the exposure blocks and one that modeled the learning blocks. Eight regressors of no interest were included to model physiological artefacts, and 6 more to model head movement. For each of the two sessions, two t-contrasts modeled the effects of the exposure and learning blocks against an implicit baseline. Over the two sessions, therefore, four contrast images were created and these were entered into the second level group analysis.

Second level analysis. At the second level, a 2 × 2 factorial ANOVA with factors of Session (with levels Basque and Control), and Condition (levels Exposure and Learn) was carried out on the normalized first level contrast images. The contrast of interest was the subtraction of the Learning conditions in each session: *t* = [Learn Basque − Learn Control]. This contrast tests for areas that are more active during the Learning phase of the Basque task than during the Learning phase of the Synonym task. A conjunction analysis between *t* = [Learn Basque] and *t* = [Learn Control] was also performed.

### 3.3 Correlations between performance improvement and task activation

If the cerebellum acquires and stores associations between the English words and their Basque translation, we expect that more recruitment of these regions will be associated with better retention of the learned information. To test whether this was the case, we correlated the BOLD response in cerebellar areas identified by the [Learn Basque − Learn Control] contrast with a measure of in-scanner learning and a measure of early consolidation (or off-line performance improvement). We define in-scanner (or on-line) learning as the difference in performance between the first and the last in-scanner test. This measure captures performance improvements over the active learning trials, where participants performed multiple-choice responses and received feedback on their answers. We define early consolidation as the performance difference between the last performance measure in the scanner and the post-scan assessment about 20 min later. This measure captures off-line performance improvements, occurring over the interval between the learning task and the later assessment. Following our hypothesis, we tested for a relation between these performance measures and the mean BOLD signal in any right cerebellar clusters found, and used a Bonferroni correction to adjust for the number of cerebellar hypotheses tested. To assess the specificity of these associations, we also performed additional exploratory analyses. Task-related activations in other brain areas were also correlated with on-line learning and off-line performance improvement. These exploratory analyses give some sense of the specificity of any correlation with the cerebellum, but cannot be functionally interpreted as they are not corrected for multiple comparisons. Note that that there is no risk of circular analysis. Circularity arises when there is a dependence between the criterion for selecting a region of interest and the measure with which it is related (Kriegeskorte, Simmons, Bellgowan, & Baker, 2010). Here, the criterion for selecting the ROIs was whether the region was more active in the Basque session than in the control session. The activation level during the Basque task was then correlated with the performance improvement during and following the Basque task. Pearson’s correlation analyses were performed in R.

## 4 Results

### 4.1 Behavioral results

**Fig. 2:**
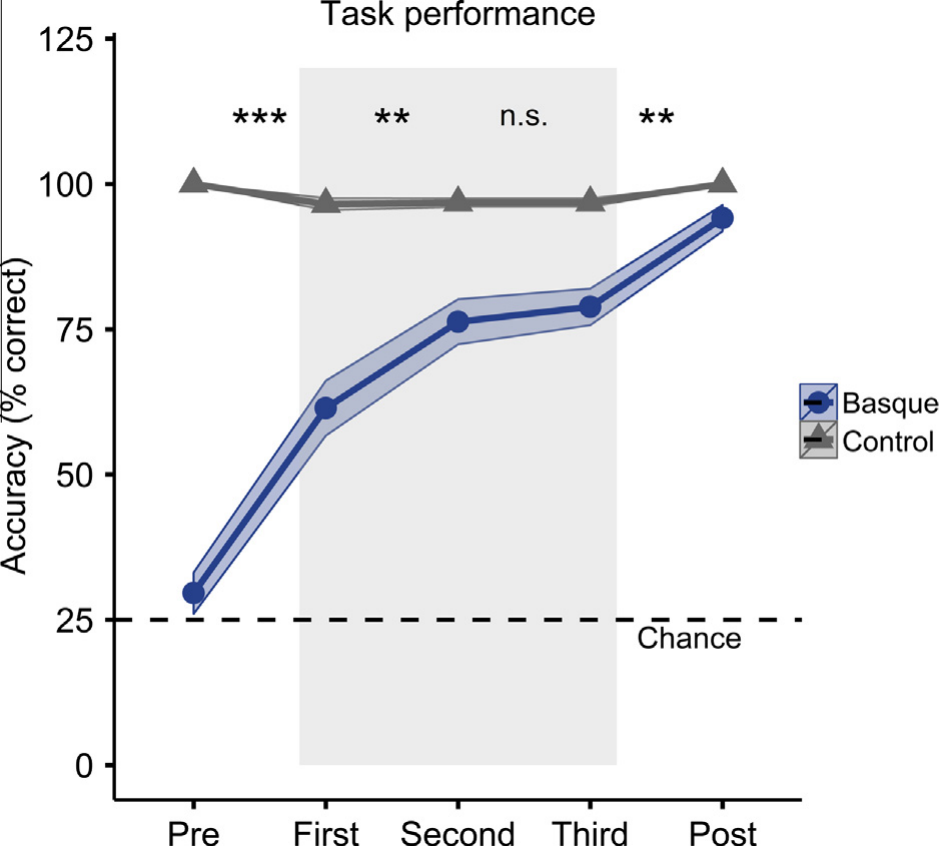
Mean task performance in the Basque (Blue) and Synonym (Gray) tasks. Dashed line represents chance level (25%). Shaded regions around each line represent ±1 standard error of the mean. Gray shaded rectangle denotes the three performance measures recorded in the MRI scanner. Dashed line indicates chance performance. Asterisks denote the significance levels of Wilcoxon rank tests testing for differences between different time points on the difference between Synonym and Basque performance. ^∗∗∗^: *p* < 0.001, ^∗∗^: *p* < 0.01, and n.s.: no significant difference.

All participants showed a clear increase in performance on the Basque task and a consistently very high performance in the synonym task (see Fig. 2). Performance on the control task was error-free in the pre-test and the post-test, and was very high and showed little variability during the scanner task. In the Basque task, performance increased from a level slightly above chance (mean = 29%, SE = 3.6%, one-sample *t*-test against chance (25%): *t*(14) = 1.336, *p* = 0.203) in the pre-test to near-perfect performance on the post-test (mean = 94%, SE = 2.2%). In order to test for a time-by-condition interaction, non-parametric tests were carried out on the difference between the scores for the synonym and Basque test. A Friedman’s ANOVA showed that this difference changed significantly over time (*χ*^2^(4) = 47.3, *p* < 0.001). Follow-up Wilcoxon rank tests show that performance increased significantly at each time point apart from the final one within the learning phase in the scanner (see Fig. 2). These results confirm that learning occurred in the Basque, but not in the control condition.

### 4.2 FMRI results

Two contrasts were carried out on the task-related components of the study: a conjunction between the Basque and control tasks and a t-contrast exposing areas more active during the Basque task than the control task. Both these contrasts concern the learning phase, and not the exposure phase.

#### 4.2.1 Areas active in the Basque and control tasks (conjunction analysis)

**Fig. 3:**
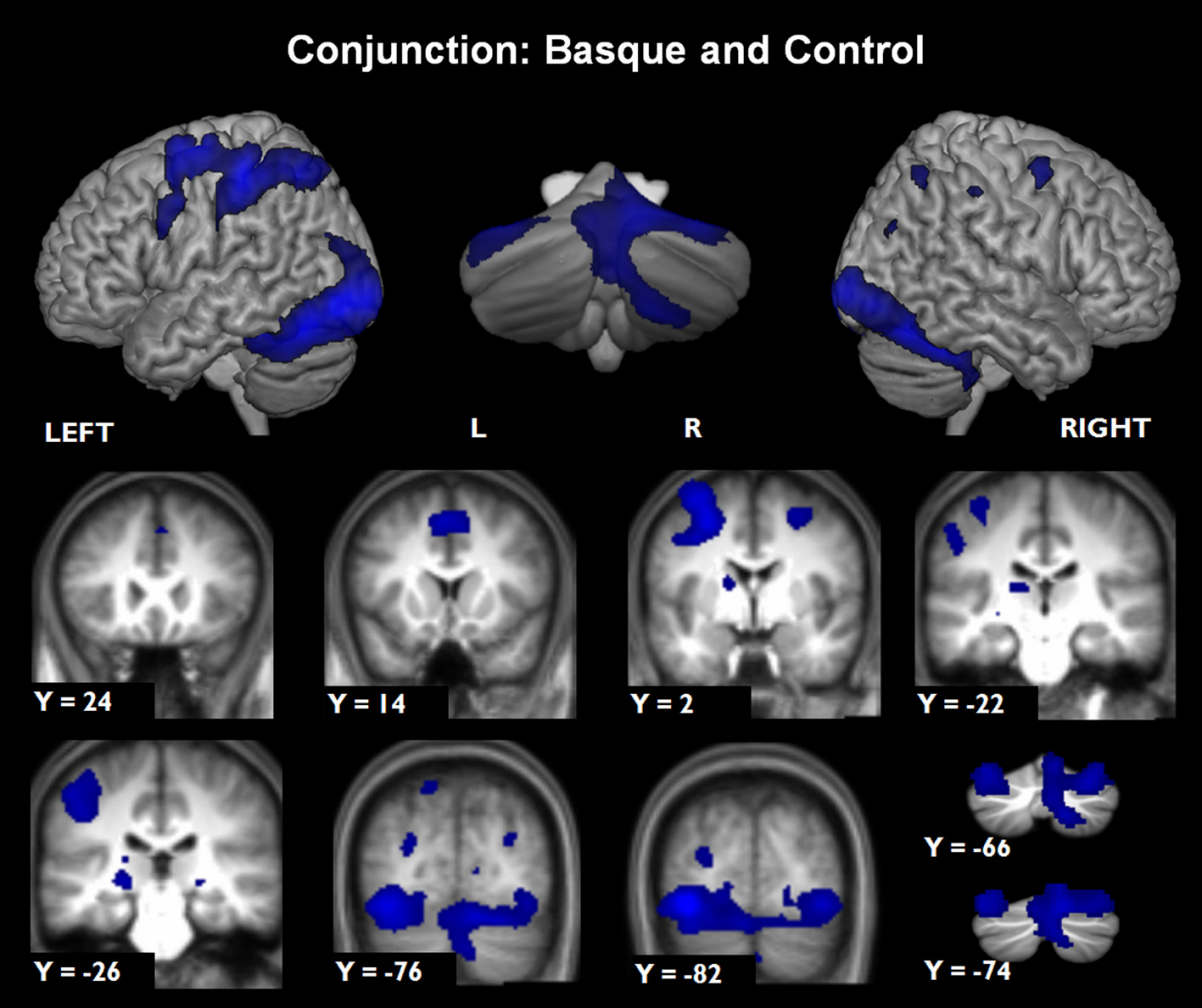
Imaging results for the conjunction analysis between the Basque learning task and the Control task. All clusters voxel-wise FWE corrected at *p* < 0.05. Surface-rendered images are projected onto the MNI-Colin27 template (http://www.bic.mni.mcgill.ca/) for whole brain, and SUIT template for cerebellum. Coronal slices are displayed on the average normalized structural images from the 15 participants, and on the SUIT template for the cerebellar slices. Left is displayed on the left.

The conjunction analysis revealed areas commonly activated in learning phases of both the Basque learning and the synonym control task. In both tasks, participants processed written language, performed a multiple choice task, and responded with finger presses of the right hand. Left-lateralized activity was found in motor and premotor cortex, with the activation extending into and covering large areas of the posterior parietal cortex. On the right, smaller activations were present in motor cortex and posterior parietal cortex. There was widespread activation bilaterally in ventral higher order visual areas. Other clusters were found in the supplementary motor area, the left anterior insula and left caudate nucleus. Cerebellar activity was noted bilaterally in the cerebellar vermis and lobule HVI, and on the right in lobule HVIII (see Fig. 3 and Table 1A).

#### 4.2.2 Areas more active during Basque learning than control task (t-contrast)

**Fig. 4:**
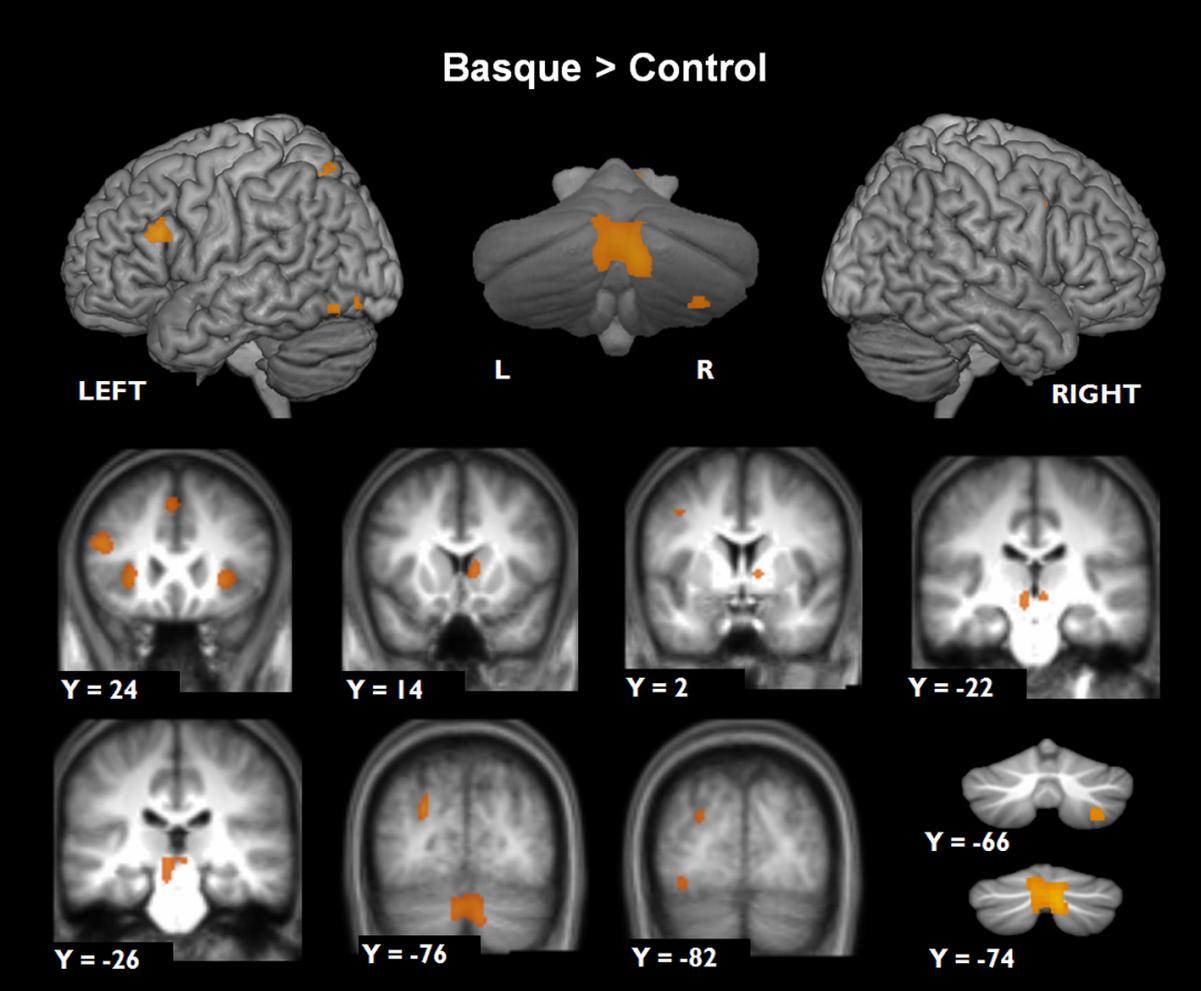
Areas more active during the Basque learning task than the Control task. All clusters voxel-wise FWE corrected at *p* < 0.05. Surface-rendered images projected onto the MNI-Colin27 template (http://www.bic.mni.mcgill.ca/) for whole brain, and SUIT template for cerebellum. Coronal slices are displayed on the average normalized structural images from the 15 participants, and on the SUIT template for the cerebellar slices. Left is displayed on the left.

The contrast of interest compared the two learning phases and exposed regions which were more active during Basque learning than during the control task with English synonyms (Fig. 4 and Table 1B). Bilateral activations were present in the anterior insula (frontal operculum), the thalamus and the cerebellar vermis. Left-lateralized activations were present in BA45, pre-SMA, superior parietal lobule and BA6. Consistent with our hypothesis, cerebellar activation was identified in right-cerebellar Crus II, and a second, larger cluster was found in the cerebellar vermis.

### 4.3 fMRI results: correlations between brain and behavioral measures

Pearson correlation analyses were carried out between cerebellar BOLD signal activations measured during the learning phase of the Basque session and two behavioral measures: in-scanner learning (improvement between first and last in-scanner assessment) and off-line improvement (early consolidation, between the last in-scanner test and the post-test, see Fig. 2). Because analyses were carried out on two cerebellar clusters and two behavioral measures, correlations were corrected for four comparisons (Bonferroni-corrected alpha <0.0125). To address the specificity of these correlations, other activated regions were also correlated with on-line and off-line performance improvement.

**Fig. 5:**
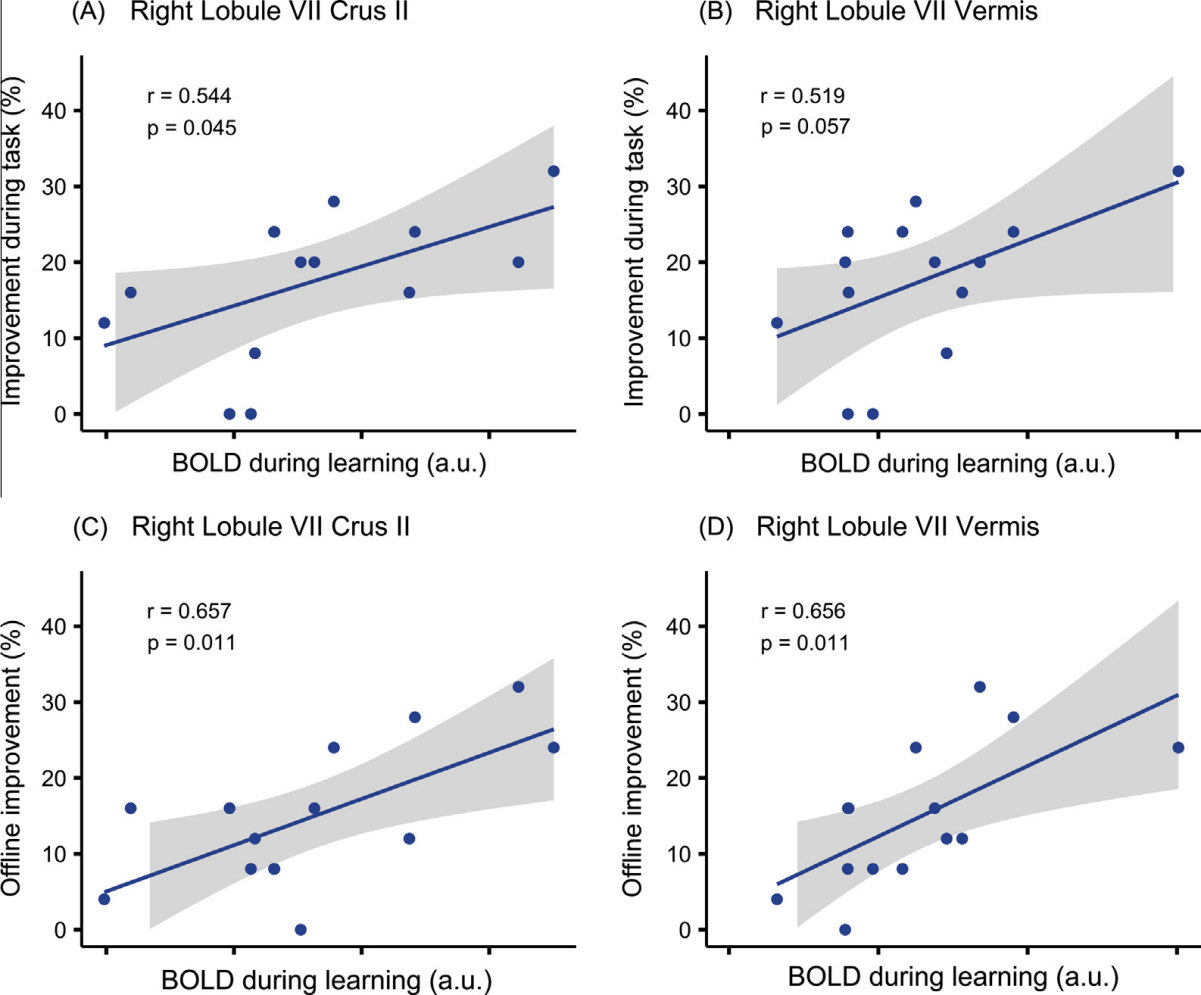
Scatter plot of in-scanner, on-line learning (A and B) and off-line performance improvement (C and D) and haemodynamic response in the Basque task in a Crus II cluster (left panels) and a cluster in the posterior vermis (right panels). Lines show fitted linear regression and shaded areas indicate confidence intervals across the group of 14 participants (±1SE). All p-values are Bonferroni-corrected alpha = 0.0125 for 4 comparisons.

Results for performance gains during the task showed that the cluster in right Crus II correlated positively with on-line, in –scanner learning (Pearson’s *r* = 0.544, *p* = 0.045), while the cerebellar vermal activation correlated marginally (Pearson’s *r* = 0.519, *p* = 0.057; see Fig. 5). While accounting for about 25% of the variance in learning, these correlations were not significant after correction for multiple comparisons. Further exploratory analyses showed that the association with on-line learning was not confined to cerebellar regions, as some supratentorial brain areas, including pre-SMA (Pearson’s *r* = 0.563, *p* = 0.036) and left occipital gyrus (Pearson’s *r* = 0.654, *p* = 0.011), showed similar positive associations, and several others showed positive trends.

In contrast, our results show that off-line improvement was strongly and significantly predicted by the BOLD signal in both the right Crus II cerebellar cluster (Pearson’s *r* = 0.657, *p* = 0.011) and the cluster in the cerebellar vermis (Pearson’s *r* = 0.656, *p* = 0.011, see Fig. 5), accounting for about 43% of the variance in off-line improvement. Exploratory analyses indicated that none of the other activated regions were correlated significantly with early consolidation after Bonferroni correction; the pre-SMA showed a positive association with early consolidation (Pearson’s *r* = 0.553, *p* = 0.040), but no other trends were observed (all Pearson’s *r* < 0.37, *p* > 0.25; see Table 2).

Thus, performance gains during the task itself showed modest associations with the BOLD response in the majority of those brain areas that were more engaged in the Basque task than the control task, including the cerebellar clusters. However, these effects did not survive correction for multiple comparisons. Conversely, early consolidation was significantly predicted by the task-related activity in the cerebellar clusters, while the supratentorial brain areas considered did not significantly predict off-line improvement.

## 5 Discussion

This study investigated fMRI activity during an explicit lexical learning task. Given the recruitment of the right cerebellum in semantic association tasks (De Smet et al., 2013) and its documented involvement in motor (Jenkins et al., 1994) and cognitive learning (Balsters et al., 2013), we hypothesized that the right cerebellum – specifically right Crus I/II – would be engaged in the acquisition of novel lexical-semantic relations. Our behavioral results showed that participants showed increased performance throughout the learning task, and a post-test about 20 min after the task showed that they had retained the newly acquired associations and had further improved. The imaging results revealed that a distributed network of brain regions was more engaged during the learning of new words than when performing a control task. These regions included two cerebellar clusters; one in right Crus II (consistent with our hypothesis) and one in the vermal region of Lobule VII. Interestingly, activity in these two cerebellar clusters during vocabulary acquisition predicted off-line performance improvements measured shortly after the task.

As hypothesized, right Crus II was significantly more active when learning novel Basque words than during a control task that posed the same sensory and motor demands. The finding that the right posterior cerebellum is active during vocabulary learning is consistent with a large body of neuroimaging evidence showing that right cerebellar areas contribute to language processing. Right cerebellar activity is consistently found in contrasts that probe semantic processing, and this activation is found regardless of stimulus modality and regardless of whether a motor response is required (Fedorenko et al., 2010, Price, 2012). Resting state functional connectivity data also demonstrate that cerebral language areas such as inferior frontal gyrus, occipitotemporal regions and the inferior parietal lobule are functionally connected to posterolateral cerebellum, notably Crus I and Crus II (Bernard et al., 2012, Buckner et al., 2011, Habas et al., 2009). Thus, both functional imaging data and resting state connectivity data indicate that posterior lateral areas of the cerebellum may be part of a language processing circuit.

Conversely, the second, larger cerebellar activation in the posterior cerebellar vermis was not hypothesized. This cluster spans lobules VI–VIII and extends into the hemispheres on both sides and was also activated, albeit to a lesser degree, in the control task. Vermal lobule VII, also named the oculomotor vermis, is chiefly implicated in saccadic and smooth-pursuit eye movements (Thier, Dicke, Haas, Thielert, & Catz, 2002). Although difficult to marry with current ideas about cerebro-cerebellar connectivity and the cerebellar contribution to higher cognition (Kelly and Strick, 2003, Stoodley, 2012), the finding that the cerebellar vermis is recruited in a linguistic learning task is not completely anomalous. In patient and imaging studies, haemodynamic activity in the vermis has been linked to emotional processing rather than language or working memory tasks (E et al., 2012, Schmahmann and Sherman, 1998, Stoodley and Schmahmann, 2009a, Timmann et al., 2010). However, the vermis has also been implicated in working memory and language tasks. This may explain the region’s strong activation in the current task, as well as the correlation with off-line performance improvement, as working memory capacity is strongly and consistently related to second language learning and proficiency (Linck, Osthus, Koeth, & Bunting, 2014). Early neuroimaging studies report both posterior vermal and right posterolateral cerebellar activations in relation to language and verbal working memory tasks (Desmond and Fiez, 1998, Fiez and Petersen, 1998, Fiez et al., 1996), and there is voxel-based morphometric (VBM) evidence that gray matter density in the vermis correlates with working memory measures (Ding, Qin, Jiang, Zhang, & Yu, 2012). Moreover, it is not uncommon for children to develop language problems, such as mutism and agrammatic symptoms following the resection of a tumor in the vermis (Riva & Giorgi, 2000). The portion of posterior right cerebellum adjacent to the vermis is often activated in semantic tasks (Devlin et al., 2000, Fedorenko et al., 2010), and in a resting state functional connectivity study, a Crus II cluster with its medial portions bordering the vermis was functionally connected to the left executive control network (Habas et al., 2009). However, these linguistic and working memory studies that show BOLD activations in the vermis and paravermis (Devlin et al., 2000, Fedorenko et al., 2010, Habas et al., 2009) tend not to have the activity peak in the vermis. In a longer-term lexical training study by Raboyeau et al. (2004) increased activity was found in the cerebellar vermis and Crus II after lexical training, with the vermal increase predicting retention two months later. Although the timescale that those authors considered vastly differs from the one in the present study, they too found that the vermis was more active during word learning and that its activity predicted future performance.

On their own, the cerebellar activations found in this study could also be explained by processing novel articulatory features, by co-activation with connected supratentorial language regions, or by any number of other processes unrelated to lexical learning. In particular, one might ascribe the increased metabolic demands on the oculomotor vermis during the processing of novel words to an increase in the number of saccades or to attentional effects, as these have been shown in early visual cortex (Twomey, Kawabata Duncan, Price, & Devlin, 2011). However, activity in Crus II as well as the posterior vermis positively predicted off-line performance improvement, a proxy for early consolidation. The more these cerebellar regions were recruited during the task, the more performance increased between the end of the task and the post-test 20 min later. Such associations were not found with any of the learning-related neocortical activations. This finding argues against co-activation of the cerebellum due merely to its connections with language regions, and against activations related to articulatory processing, increased eye-movements or response preparation. None of these alternative accounts would predict cerebellar activity to be related to a measure of early consolidation, or at least not without similar relationships being seen in supratentorial recruited regions. Thus, the present data suggest that whichever processes generate the increased haemodynamic response in the cerebellum during learning, they are associated with the successful acquisition and retention of new words.

The pattern of cerebral activations during the learning vs. control condition are consistent with results of previous studies of word learning. Learning-related activity was found in left inferior frontal gyrus (BA45), bilateral insula, pre-SMA, left premotor cortex (BA6), left superior parietal cortex, right caudate, the right cerebellar vermis and in right Crus II. The clusters in posterior parietal cortex, occipital cortex and the cerebellar vermis were also active in the conjunction analysis of the learning and the control task. This suggests that these regions subserve functions that are necessary for visually processing known associations and making responses accordingly, but are recruited to a greater extent when encoding novel lexico-semantic associations. Previous PET and fMRI data into verbal encoding and explicit learning tasks consistently implicates inferior frontal cortex (Buckner et al., 1999, Kapur et al., 1994, Kapur et al., 1996). Other areas are reported inconsistently in word learning paradigms, which is likely due to the large variability in task type, timescale and modality. In a study by Davis et al. (2009) novel words learned just prior to scanning elicited more activity in inferior frontal and premotor cortex, left superior temporal gyrus and right cerebellum than familiar words. Breitenstein et al. (2005) looked at change over time in a picture-word associative learning task and report declining activity in the hippocampus and fusiform gyrus, and increasing activity in angular gyrus. Activations in anterior insula and SMA/pre-SMA have been associated with the phonological processing of new words (Paulesu, Frith, & Frackowiak, 1993), and pre-SMA receives input from non-motor domains of the cerebellum (Akkal, Dum, & Strick, 2007). The left premotor cortex is mainly concerned with the preparation of movements, but it has also been implicated in the rule-based association of symbolic cues (Hanakawa et al., 2002). The right caudate nucleus, which was more active during the learning task, is implicated in various semantic and phonological tasks (Abdullaev, Bechtereva, & Melnichuk, 1998). Activation of the left ventral occipital cortex is commonly found in tasks using written verbal material (Price and Devlin, 2011, Price and Mechelli, 2005). This area was also robustly activated in the conjunction analysis and the increased attention to the written words may explain the further increased BOLD response during the learning task (Twomey et al., 2011). In addition, this region is also consistently implicated in artificial grammar learning tasks, suggesting a role in language learning (Opitz and Friederici, 2003, Petersson et al., 2012). Interestingly, the exploratory correlation analyses revealed a relationship between the recruitment of the occipital (BA19) cluster during the learning task and performance improvement throughout the task. Because this relationship was not hypothesized, we cannot interpret this correlation at this time. However, future investigations into lexical learning can address whether the ventral occipital activation is consistently associated with improved learning performance.

Seemingly at odds with previous findings, our results for the learning task show no activations in either hippocampus, or left temporal neocortical regions. The former structure is deemed critical for semantic learning and the latter regions are thought to store lexical and semantic knowledge (Binder et al., 2009, Patterson et al., 2007, Price, 2012). However, imaging studies of word learning do not consistently find hippocampal activity, and there are indications that hippocampal activity assessed with fMRI is only evident when stimuli are entirely novel to the participant (Dolan & Fletcher, 1997). This was not the case in the present study, where an exposure phase preceded the learning phase. Moreover, activity in superior and middle temporal gyrus is typically found in paradigms using auditory stimuli, and may therefore in part reflect audition-specific learning (Breitenstein et al., 2005, Davis et al., 2009, Dobel et al., 2010, Shtyrov et al., 2010). Given the visual nature of the stimuli, one might then expect more widespread activation in the posterior inferior temporal cortex, which is not the case here. However, early PET studies looking at the encoding of visually presented verbal stimuli do not report neocortical temporal activations either (Kapur et al., 1994, Kapur et al., 1996). A further explanation could be that the temporal association cortex becomes more engaged in later stages of learning than during encoding per se. Right cerebellar activity is reported in some of these previous studies, which span a variety of designs with different stimulus modalities and time scales (Breitenstein et al., 2005, Davis et al., 2009, Raboyeau et al., 2004).

This study has some limitations that constrain the scope of inference. First, our sample size was relatively modest with 15 participants each scanned twice. Though we were able to identify robust responses related to lexical learning, this study may not have had enough power to detect more subtle effects. Second, our design was block-related, and we were therefore unable to model trial-by-trial learning, or to consider erroneous responses separately from correct responses.

A large body of evidence implicates that cerebellum in cognitive (Hayter et al., 2007, Lesage et al., 2010, Marvel and Desmond, 2010, Pope and Miall, 2012) and language (Mariën et al., 2013, Stoodley and Schmahmann, 2009b) function, but there is presently no consensus about what the cerebellar contribution to these higher-order processes may be. One prominent theoretical framework ascribes the cerebellum a fundamentally predictive role in motor control, whereby short-term predictions of upcoming sensory and proprioceptive stimuli enable smooth, coordinated movements and adaptive control. These predictions are generated by internal models of motor operations, which are acquired through learning (Miall, Weir, Wolpert, & Stein, 1993). There is much evidence for forward model prediction in motor control and motor learning (Kawato et al., 2003, Miall, 1998, Wolpert and Miall, 1996, Wolpert et al., 1998), and given the striking cytoarchitectonic homogeneity of the cerebellum, it has been proposed that such forward models are also present in the cerebellar regions that govern cognitive and linguistic operations (Argyropoulos, 2011, Ito, 2008, Ramnani, 2006). Interestingly, in the field of the neurobiology of language processing several forward modeling accounts have recently been proposed (Kotz and Schwartze, 2010, Rothermich and Kotz, 2013, Tian and Poeppel, 2010). Particularly relevant for the task in this study, Pickering and Garrod, 2014, Pickering and Garrod, 2013 recently proposed that forward model processes may underlie language comprehension and production at semantic, phonological and orthographic levels. Consistent with the notion of cerebellar forward model prediction in language comprehension, we have previously demonstrated a disruption of performance in a semantic prediction task following right cerebellar rTMS, that is consistent with a role for the right posterolateral cerebellum in semantic prediction (Lesage, Morgan, Olson, Meyer, & Miall, 2012). Speculatively, such prediction may be subserved by knowledge of semantic associations, the representations of which may be acquired by and stored in the right cerebellum. While the current study does not directly address this hypothesis, the results are consistent with this notion. Our study is the first to specifically address a cerebellar contribution during a short-term explicit vocabulary learning paradigm. Our findings demonstrate that the cerebellum plays a role in the early consolidation of an explicitly learned lexicon. Future investigations can address whether this also holds true for vocabulary that is acquired incidentally, and whether the cerebellum is implicated in longer-term consolidation processes. Further, the robust activation in the cerebellar vermis was unexpected, and it will be interesting to see if this region proves consistently active during different language learning and working memory tasks.

In conclusion, the present results provide further evidence for a right cerebellar role in language, and we provide strong evidence for its role in the acquisition of a novel lexicon, whereby cerebellar activity during the learning task was predictive of off-line performance gains following the task.

## Figures and Tables

**Table 1:** Tables of results. A. Areas commonly activated in the Basque and Control tasks. B. areas more active in Basque learning task than in Control task (FWE corrected; minimum cluster size 200 mm^3^ voxels).

Contrast	Gross anatomical location	Volume (mm^3^)	*T*-value	MNI coordinate			Cytoarchitectonic region
				*x*	*y*	*z*	
*Conjunction Basque and Control*							
Left Inferior Occipital Gyrus		149,984	13.85	−36	−82	−8	BA19
	Left Middle Frontal Gyrus		9.97	−30	−2	52	BA8
	Left Precentral Gyrus		10.09	−38	−6	66	BA6
	Left Postcentral Gyrus		11.78	−46	−34	52	BA2
	Left Superior Parietal Lobule		10.4	−28	−60	52	BA7
	Left Inferior Occipital Gyrus		13.85	−36	−82	−8	BA19
	Left Fusiform Gyrus		12.36	−38	−64	−18	BA19/BA37
	Left Inferior Occipital Gyrus		11.73	−20	−96	−6	BA19
	Left Inferior Temporal Gyrus		11.29	−42	−42	−18	BA37
	Right Cerebellum		11.17	32	−52	−25	Lobule HVI
	Right Cerebellum		10	18	−60	−47	Lobule HVIII
Right Middle Frontal Gyrus		2600	7.47	30	2	54	BA8
Right Superior Parietal Lobule		2368	6.91	30	−58	52	BA39
Right Middle Occipital Gyrus		808	6.83	32	−70	28	BA19
Left caudate nucleus		2480	7.6	−14	−8	14	n/a
Left Cerebellum		712	5.85	−22	−34	−43	Lobule HX
Right Intraparietal Cortex		384	5.98	48	−30	46	n/a
*Basque > Control*							
Left Middle Occipital Gyrus		2768	7.1	−26	−74	30	BA19
	Left Superior Parietal Lobule		6.64	−24	−68	46	BA7
Right Cerebellum		4712	6.52	8	−74	−35	Lobule VII (vermis)
Left Inferior Frontal Gyrus		1784	6.92	−48	22	24	BA45
Left thalamus		1192	6.52	−6	−24	−4	n/a
	Right thalamus		6.03	6	−22	−2	n/a
Right caudate Nucleus		1024	6.28	12	2	2	n/a
Right anterior insular cortex		984	6.72	30	26	0	n/a
Left Inferior Occipital Gyrus		760	6.14	−48	−70	−14	BA19
Left anterior insular cortex		672	6.59	−32	26	2	n/a
Left pre-SMA		512	6.17	−2	22	48	BA6
Right Cerebellum		376	5.82	32	−66	−53	Lobule HVII Crus II

**Table 2:** Correlation between task activations and off-line performance improvement. (Bonferroni corrected alpha *p* < 0.0125). ^+^ indicates *p* < 0.05 (uncorrected for multiple comparisons), ^∗^ indicates *p* < 0.0125 (corrected for 4 comparisons).

Anatomical location	MNI coordinate			Correlation: in-scanner		Correlation: consolidation	
	*x*	*y*	*z*	Pearson’s *r*	*p*	Pearson’s r	*p*
Right Cerebellum (Vermis)	8	−74	−35	0.519	0.057	0.656	0.011^∗^
Right Cerebellum (Crus II)	32	−66	−53	0.544	0.045^+^	0.657	0.011^∗^
Left Inferior Frontal Gyrus	−48	22	24	0.273	0.344	0.326	0.255
Left pre-SMA	−2	22	48	0.563	0.036^+^	0.553	0.040^+^
Left Superior Parietal Lobule	−26	−68	46	0.469	0.091	−0.092	0.753
Left Inferior Occipital Gyrus	−48	−70	−14	0.654	0.011^∗^	0.046	0.875
Right frontal insular cortex	30	26	0	0.478	0.084	0.324	0.259
Left frontal insular cortex	−32	26	2	0.278	0.336	0.378	0.183
Right Caudate Nucleus	10	14	6	0.468	0.092	0.329	0.251
