# Supplementary material for: Cerebellar BOLD signal during the acquisition of a new lexicon predicts its early consolidation
Source: Brain Lang. 2016 Oct;161:33–44. doi: 10.1016/j.bandl.2015.07.005 (PMC5066914; doi:10.1016/j.bandl.2015.07.005)
Supplement: Supplementary data 1 [file mmc1.docx]

## Appendix: Stimuli used in the vocabulary learning task

| Basque |  | Synonym |  |
| --- | --- | --- | --- |
| Stimulus | Target | Stimulus | Target |
| laranjak | oranges | glasses | specs |
| behia | cow | ball | sphere |
| loreak | flowers | bush | shrub |
| haurra | baby | knife | scalpel |
| jantzi | dress | adhesive | glue |
| hartza | bear | chair | seat |
| aterki | umbrella | dog | hound |
| logela | bedroom | field | meadow |
| gazta | cheese | bag | satchel |
| baso | forest | truck | lorry |
| arkatza | pencil | monitor | screen |
| anaia | daughter | scent | odour |
| tapiza | rug | money | cash |
| zorro | wallet | picture | image |
| arrain | fish | tin | can |
| horma | wall | cylinder | tube |
| izarrak | stars | twig | branch |
| katilua | cup | stream | brook |
| aita | father | house | home |
| sagua | mouse | fire | blaze |
| elurra | snow | author | writer |
| ogia | bread | woman | lady |
| otsoa | wolf | sea | ocean |
| leiho | window | bloke | guy |
| koilara | spoon | frock | gown |
